# Fibroblast growth factor 21 ameliorates behavior deficits in Parkinson's disease mouse model via modulating gut microbiota and metabolic homeostasis

**DOI:** 10.1111/cns.14302

**Published:** 2023-06-19

**Authors:** Changwei Yang, Wuqiong Wang, Pengxi Deng, Xinyi Wang, Lin Zhu, Liangcai Zhao, Chen Li, Hongchang Gao

**Affiliations:** ^1^ Institute of Metabonomics & Medical NMR, School of Pharmaceutical Science Wenzhou Medical University Wenzhou China; ^2^ School of Public health Fujian Medical University Fuzhou China; ^3^ Oujiang Laboratory (Zhejiang Lab for Regenerative Medicine, Vision and Brain Health) Wenzhou China

**Keywords:** FGF21, metabolism, microbiota–gut–brain metabolic axis, neurotransmitter, Parkinson's disease

## Abstract

**Aims:**

The effects of FGF21 on Parkinson's disease (PD) and its relationship with gut microbiota have not been elucidated. This study aimed to investigate whether FGF21 would attenuate behavioral impairment through microbiota–gut–brain metabolic axis in 1‐methyl‐4‐phenyl‐1,2,3,6‐tetrahydropyridine (MPTP) induced PD mice model.

**Methods:**

Male C57BL/6 mice were rendomized into 3 groups: vehicle (CON); MPTP 30 mg/kg/day i.p. injection (MPTP); FGF21 1.5 mg/kg/d i.p. injection plus MPTP 30 mg/kg/day i.p. injection (FGF21 + MPTP). The behavioral features, metabolimics profiling, and 16 s rRNA sequencing were performed after FGF21 treatment for 7 days.

**Results:**

MPTP‐induced PD mice showed motor and cognitive deficits accompanied by gut microbiota dysbiosis and brain‐region‐specific metabolic abnormalities. FGF21 treatment dramatically attenuated motor and cognitive dysfunction in PD mice. FGF21 produced a region‐specific alteration in the metabolic profile in the brain in ways indicative of greater ability in neurotransmitter metabolism and choline production. In addition, FGF21 also re‐structured the gut microbiota profile and increased the relative abundance of *Clostridiales*, *Ruminococcaceae*, and *Lachnospiraceae*, thereby rescuing the PD‐induced metabolic disorders in the colon.

**Conclusion:**

These findings indicate that FGF21 could affect behavior and brain metabolic homeostasis in ways that promote a favorable colonic microbiota composition and through effects on the microbiota–gut–brain metabolic axis.

## Graphical abstract

FGF21 attenuated the motor and non‐motor dysfunction in PD mice. FGF21 regulated choline metabolism, glutamate‐glutamine cycle, and energy metabolism in the brain. FGF21 treatment restores PD‐induced shifts of colonic microbiota and modulates colon metabolism.

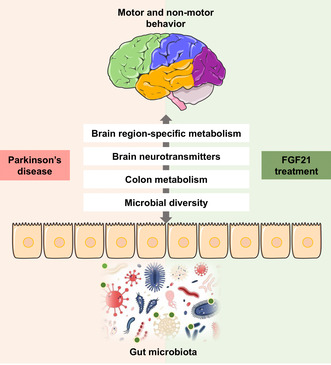

## 1 BACKGROUND

Parkinson's disease (PD), which affects about 10 million people globally, is the second most prevalent neurodegenerative disease worldwide. The patient's quality of life is significantly impacted by both motor symptoms like rigidity and resting tremor as well as non‐motor symptoms like depression and cognitive dysfunction.^1^ Unfortunately, the benefits of current PD therapies are limited to motor symptoms, with little impact on non‐motor symptoms. Previous studies have found that PD‐induced motor and non‐motor symptoms are connected to neurotransmitter metabolism, mitochondrial dysfunction, and neuroinflammation.^2^, ^3^, ^4^

Recently, metabolic disorders have been considered to contribute to the development of neurodegenerative diseases including PD^5^ and Alzheimer's disease (AD).^6^ In patients with PD, for example, reduced energy metabolism and elevated levels of lactate in the brain have been identified.^7^ In our previous study, proton nuclear magnetic resonance (^1^H NMR) based metabolomics was applied to evaluate the metabolic signatures in different stages of PD, and results suggest that the overactive glutamate–glutamine metabolic cycle in the striatum was linked to the dopaminergic neuronal loss and motor dysfunction in PD mice.^8^, ^9^ Furthermore, emerging evidence has revealed that disturbed gut microbiota may also significantly contribute to the pathogenesis of PD.^10^, ^11^ And, through the microbiota–gut–brain axis, metabolic crosstalk between the gut and brain may be one of the links between gut microbiomes and brain function. As a result, novel therapeutic approaches targeting the microbiota–gut–brain metabolic axis could provide new ways to slow the progression of PD.

Fibroblast growth factor 21 (FGF21), a new member of the fibroblast growth factor family, is a recently discovered endocrine hormone primarily expressed in the liver and exerts diverse effects on metabolic health.^12^, ^13^ Tyrosine kinase FGF receptor (FGFR) and β‐Klotho in the target cell form a cell surface receptor complex that binds to FGF21 to trigger FGFR signaling activity.^14^ Previous studies have shown that FGF21 may directly affect brain function through metabolic regulation,^15^ in addition to its effects on energy consumption.^16^ The findings that FGF21 protects against obesity‐induced cognitive dysfunction^17^ and ameliorates neurodegeneration in rats and cellular models of AD highlight the value of FGF 21 in brain health.^18^ Our previous study has shown that FGF21‐attenuated neurodegeneration in cellular and mouse models of PD modulating microglia polarization.^19^ Given the safety record of human clinical trials, FGF21 could be a promising therapy for clinical trials for patients with PD. However, more direct evidence is still needed to elucidate the therapeutic effects of FGF21 against PD.

More importantly, as a classic metabolic regulator, the effects of FGF21 on PD and its relationship with gut microbiota have not been elucidated. The current study addressed this issue by investigating the potential effect of FGF21 on metabolic homeostasis in the brain and colon in PD mice. Moreover, the molecular insights connected with the microbiota–gut–brain axis in the beneficial role of FGF21 in PD were discussed as well.

## 2 METHODS

### 2.1 MPTP and FGF21 administration

Male C57BL/6 mice (20–26 g), were purchased from the SLAC Laboratory Animal Co. Ltd. Shanghai, China, and then maintained in the laboratory animal center of the Wenzhou Medical University under regular laboratory conditions. A previous study showed that males of at least 8 weeks of age weighing at least 22 g are the ones that produced the most consistent results in MPTP induced PD mice model.^20^ 1 week later, all of the mice were randomly assigned to one of the three groups (*n* = 10 in each group): CON, MPTP, and FGF21 + MPTP groups. Mice in the MPTP and FGF21 + MPTP groups were injected intraperitoneally (i.p.) 30 mg/kg MPTP–HCl (M0896; Sigma‐Aldrich) daily for 5 consecutive days,^21^ while the mice in the CON group received an equal volume of normal saline. FGF21 was given intraperitoneally at a dose of 1.5 mg/kg/d for 7 days from the second week after MPTP treatment. Bodyweight was measured before MPTP injection and after the FGF21 treatment. Tissue samples including brain and colon were collected from mice at the time of euthanasia and stored at −80°C until analysis. All experimental protocols were approved by the Animal Ethics Committee of Wenzhou Medical University (Permission Number: wydw2018180).

### 2.2 Behavior test

Behavior performance was assessed using open field and Y maze tests before the sacrifice. The open‐field device is designed to measure rodents' spontaneous activity. Mice were tested in the open field for 5 min. Gross movement (velocity, rotations) and time spent in the different zone were measured using video‐tracking software. Additionally, the Y maze was adapted to measure cognitive function. Mice were subjected to the Y maze by placing them in the start arm and were allowed to explore freely all three arms of the maze for 5 min. The number and the sequence of arms entered were recorded and used to calculate spontaneous alteration.

### 2.3 Immunohistochemistry

Brain samples were rapidly dissected and then sectioned at a thickness of 5 μm. The paraffin‐embedded sections were then deparaffinized and rehydrated, followed by incubation with primary antibody to tyrosine hydroxylase (TH, Cell Signaling) at 4 °C for 24 h. And then incubated with a second Goat Anti‐Rabbit IgG H&L antibody, the immunoreactivity was visualized with a Nikon ECLIPSE 80i (Nikon) and then analyzed using Image J software.

### 2.4 Colonic microbiota DNA extraction and sequencing

Microbiota sequencing was conducted to determine the effects of FGF21 on PD‐induced microbiota alteration (Method S1). All DNA isolation, enrichment, and sequencing were performed on an Ion S5TM XL platform by Novogene (Beijing, China). Briefly, colonic total genome DNA was extracted using CTAB/SDS method and quantified on 1% agarose gels. The V4 hypervariable regions of 16S rRNA genes were amplified using specific primers (515F‐806R) and the indexing barcode was integrated. Single‐end reads were de‐multiplexed by barcode and quality filtering, and sequencing analyses including Alpha diversity and Beta diversity were performed using Quantitative Insights Into Microbial Ecology software (QIIME) and displayed with R software.^22^ Furthermore, Linear discriminant analysis effect size (LEfSe) analysis^23^ was conducted to identify taxa or pathways differentially abundant between CON, MPTP, and FGF21+MPTP groups.

### 2.5 NMR spectroscopy and data processing

Water‐soluble metabolites in the striatum, midbrain, cortex, and colon were identified and quantified using ^1^H NMR spectroscopy.^9^ Briefly, the frozen tissue was weighed and homogenized using an electric homogenizer with ice‐cold methanol (4 mL/g), chloroform (4 mL/g) and distilled water (2.85 mL/g). The mixture was then kept on ice for 15 min and centrifuged at 10,000 g for 15 min at 4°C. Finally, the supernatant was lyophilized and redissolved using 99.5% D_2_O with 0.05% of sodium trimethylsilyl propionate‐d4 (TSP) as the chemical shift reference. All ^1^H NMR spectra of the brain and colon were carried out on a Bruker AVANCE III 600 MHz NMR spectrometer with a 5‐mm TXI probe (Bruker BioSpin) at 298 K. Acquisition parameters were set: acquisition time, 2.65 s per scan; relaxation delay, 6 s; data points, 64 K; spectral width, 12,000 Hz. Then, all ^1^H NMR spectra were phase/baseline corrected manually using Topspin software (v2.1 pl4; Bruker BioSpin).

After removing the region of 4.60–5.20 ppm to eliminate artifacts related to the residual water resonance, the remaining spectral segments were normalized and typed in SIMCA software (v.12.0; Umetrics) for multivariate recognition analysis, including principal component analysis (PCA) and optimized potentials least squares‐discriminant analysis (OPLS–DA).

### 2.6 Statistical methods

The significance of the difference in alpha diversity measures (the observed species and Shannon index) among the 3 groups was analyzed using non‐parametric Kruskal–Wallis. Beta diversity was calculated by weighted UniFrac distances and visualized by principal coordinates analysis. In addition, Adonis, a permutational analysis of variance, was performed using 999 permutations to test for differences. In addition to microflora sequencing, the significance of group differences for normally distributed data was analyzed using a one‐way analysis of variance (ANOVA) combined with Tukey's multiple comparisons test. Data that do not exhibit a normal distribution were analyzed using the non‐parametric Kruskal–Wallis test, and data normality was tested using the Shapiro–Wilk test. Statistical analysis of group differences was carried out using SPSS for Windows 19 Inc Chicago, with a level of *p* < 0.05 accepted as statistically significant.

## 3 RESULTS

### 3.1 FGF21 ameliorates MPTP‐induced motor and non‐motor behavioral deficits in PD mice

**FIGURE 1:**
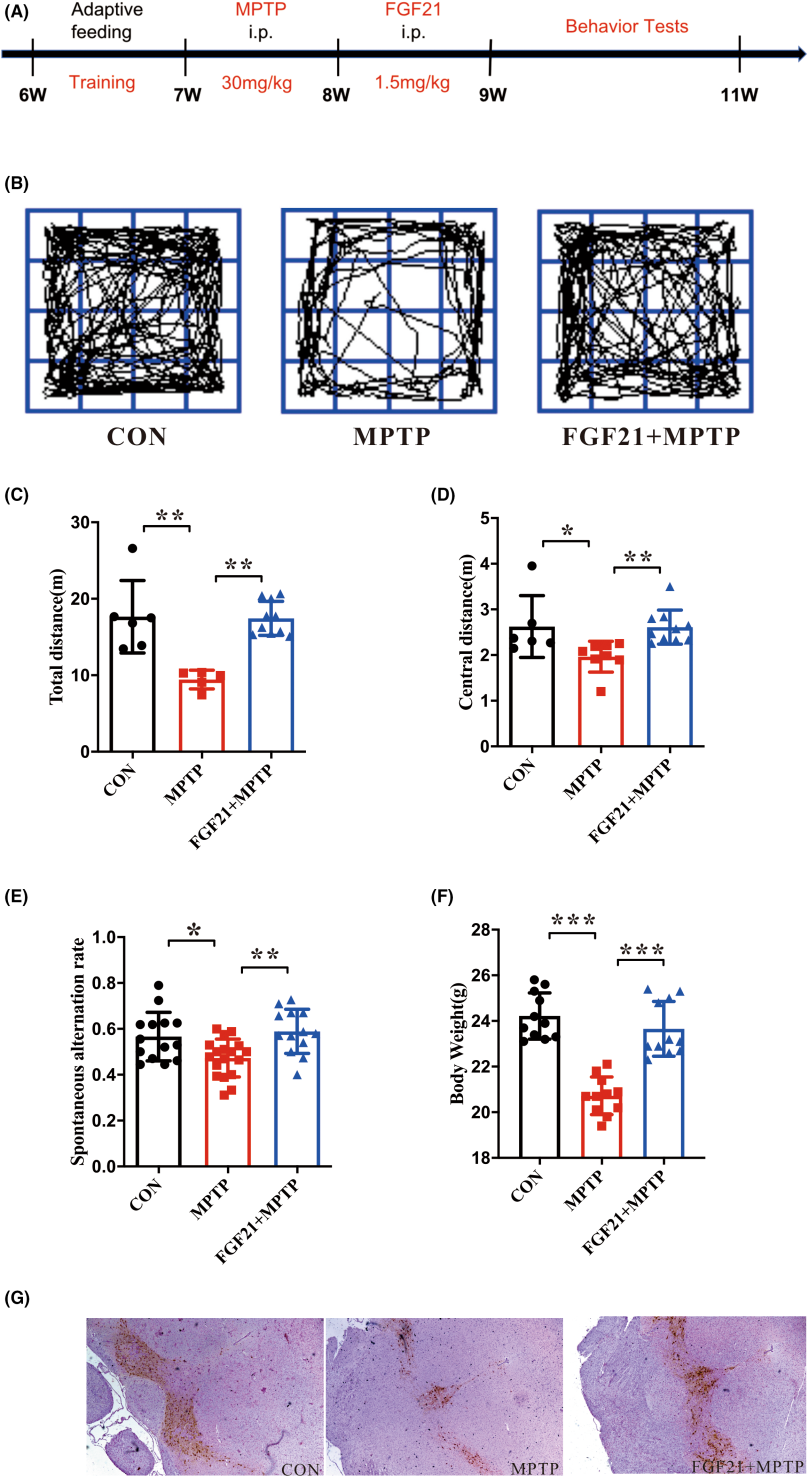
FGF21 ameliorates MPTP‐induced motor and non‐motor behavioral deficits in PD mice. (A) Flowchart of the experimental procedure. (B) Representative movement tracks. (C) Total distance traveled in the open field (*F*‐value = 13.81, df = 20, *p* = 0.0002, ANOVA test). (D) The distance traveled in the center zone of the open field (*F*‐value = 5.47, df = 21, *p* = 0.01, ANOVA test). (E) The spontaneous alternation in the Y maze (*F*‐value = 6.85, df = 44, *p* = 0.0027, ANOVA test). (F) Weight gain of mice (*F*‐value = 37.09, df = 32, *p* < 0.001, ANOVA test). (G) TH immunohistochemical staining of brain. Data were expressed as the mean ± SEM; Significant correlations are expressed as *p < 0.05; **p < 0.01; ***p < 0.001.

To assess whether FGF21 can attenuate the MPTP‐induced motor locomotion, and exploration abilities, we treated mice with FGF21 followed by MPTP injection (Figure 1A). Spontaneous locomotor activity was evaluated in the open field test, which is controlled by the nigrostriatal pathway (Figure 1B)^24^; FGF21 treatment significantly improved the total distance traveled in the open field compared with untreated PD mice (*p* < 0.001, Figure 1C). Furthermore, mice from the FGF21 + MPTP group spent slightly more time in the center of the open field, compared with PD mice (*p* < 0.05, Figure 1D). We also examined the effects of FGF21 on spatial learning and working memory using the Y Maze test. Results showed that FGF21‐treated mice had a much higher rate of spontaneous alteration than PD mice (Figure 1E). In addition, after MPTP injection, the bodyweight of PD mice was severely reduced, whereas the bodyweight of FGF21 animals was greatly increased when compared with that of PD mice (*p* < 0.001, Figure 1F). Further TH staining results showed that TH‐positive dopaminergic neuron survival in the substantia nigra was significantly increased after FGF21 treatment, compared with PD mice (Figure 1G). Taken together, these data indicate that FGF21 treatment alleviates MPTP‐induced motor symptoms and cognitive dysfunction.

### 3.2 FGF21 restores region‐specific metabolic homeostasis in the brain of PD mice

**FIGURE 2:**
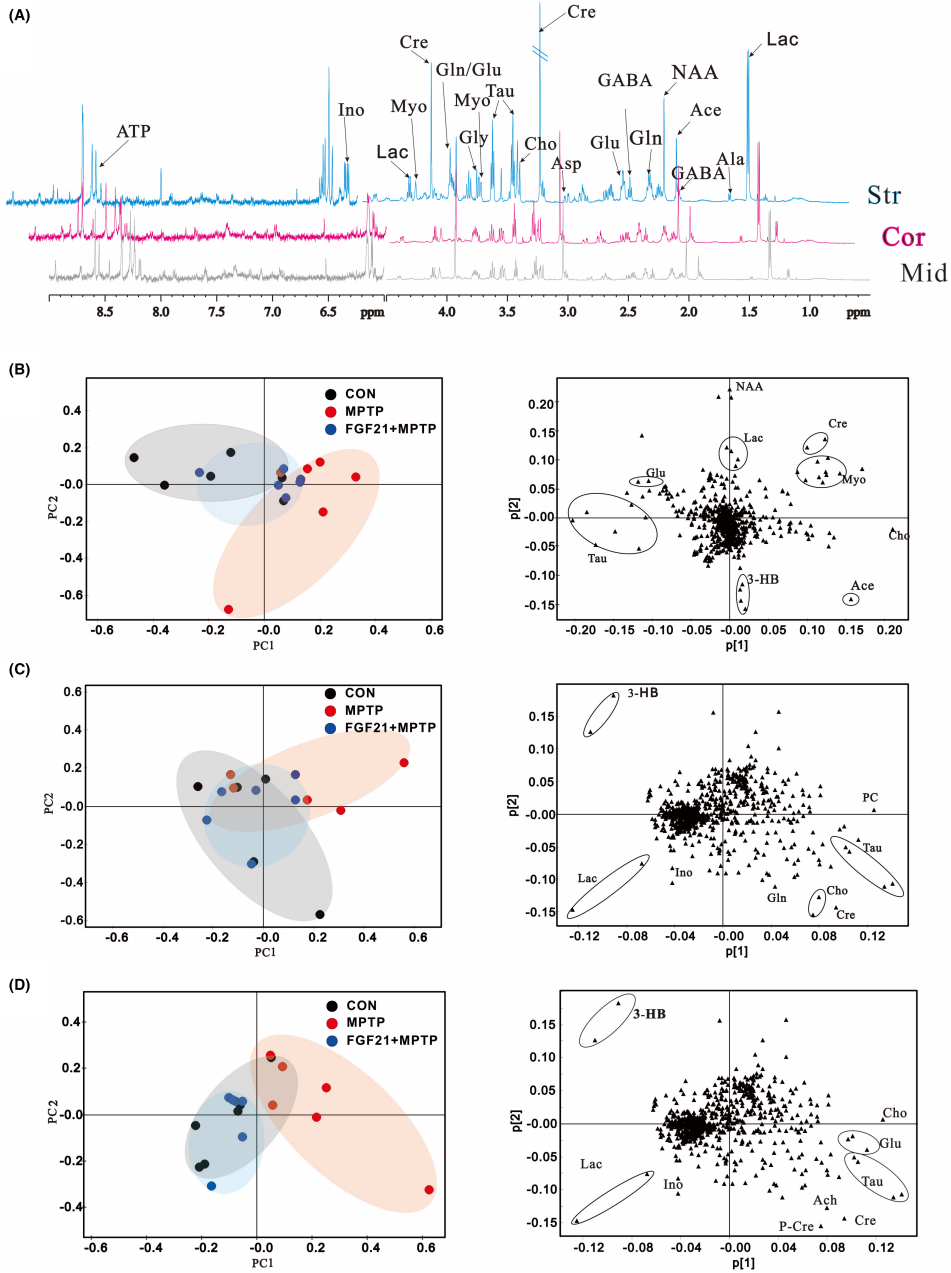
FGF21 restores region‐specific metabolic homeostasis in the brain of PD mice. (A) Representative ^1^H NMR spectra of the extracts obtained from striatum, cortex, and midbrain (*n* = 5–6 for each group). Principle component analysis (PCA) of CON, PD, and FGF21 mice in different brain regions: (B) Midbrain. (C) Striatum. (D) Cortex. 1. Lactate (Lac); 2. Alanine (Ala); 3. γ‐aminobutyric acid (GABA); 4. N‐acetyl aspartate (NAA); 5. Glutamine (Gln); 6. Glutamate (Glu); 7. Dimethylamine; 8. Aspartate (Asp); 9. Creatine (Cre); 10. Choline (Cho); 11. Glycerol‐phosphocholine (GPC); 12. Taurine (Tau); 13. Myo‐inositol (Myo); 14. Glycine (Gly); 15. Glu/Gln; 16. Inosine (Ino); 17.ADP/AMP; 18. Fumarate (Fum); 19. Adenosine monophosphate (AMP); 20. inosincacid, inosine monophosphate (IMP)

Typical ^1^H NMR spectra of the striatum, cortex and midbrain are shown in Figure 2A. The principal compoent analysis (PCA) showed that FGF21 and PD mice could be separated in a PCA score plot produced from the midbrain (Figure 2B **)**, whereas the FGF21 treated mice could not be distinguished from CON mice, especially along the first principal component PC1 (With PC1 and PC2 accounted for 32.0% and 25.6% of the total variance, respectively, Figure 2B **)**. Furthermore, the corresponding loading plot of PCA identified that the metabolic difference was ascribed to choline (Cho), Glycerol‐phosphocholine (PC), taurine (Tau), lactate (Lac), 3‐Hydroxybutyrate (3‐HB), and glutamine (Gln). Similarly, the PCA score plots of striatum (Figure 2C) and cortex (Figure 2D) also demonstrated a strong separation between the MPTP group and the FGF21 + MPTP group. Additional loading plots revealed that FGF21 protection was influenced by metabolic differences in choline metabolism, glutamate–glutamine cycle, and energy metabolism. These results were futher supported by optimized potentials least squares‐discriminant analysis (OPLS–DA), the OPLS–DA score plots revealed a clear seperation between FGF21 and PD mice (Figure S1B). As seen in the VIP score, the top contributing metabolites in the midbrain are Lac, Glu, Gln, GABA, Cre, Tau, and Myo (Figure S1A,B). Similar observations were also found in the metabolic profile in striatum and cortex between FGF21 and PD mice, with Glu, Gln, GABA, Lac, and Tau being identified as contributing metabolites in the striatum and cortex (Figure S1C–F). Together, these data suggest that FGF21 treatment induced regional‐specific changes in the profile of brain metabolites.

**FIGURE. 3:**
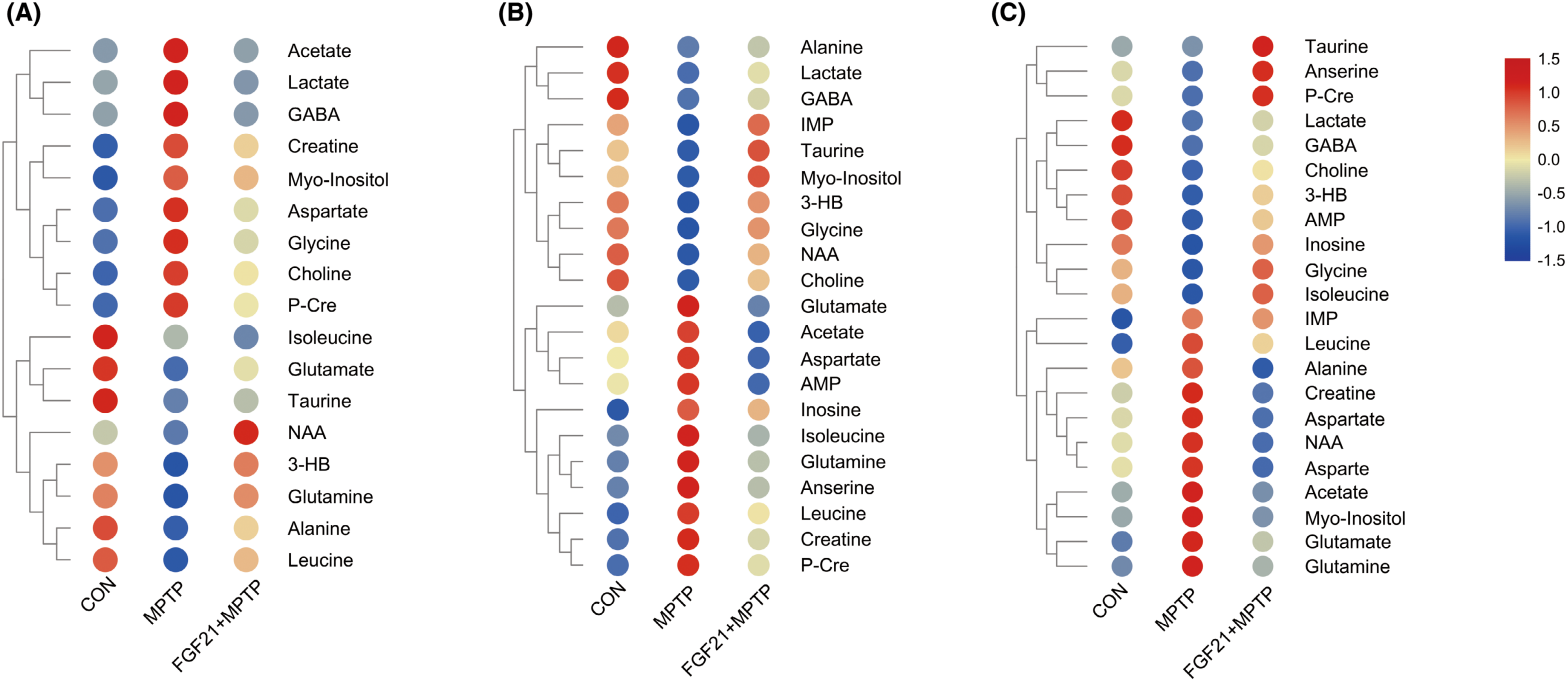
Effects of FGF21 on brain metabolites in PD mice. Brain metabolites whose concentrations are impacted by FGF21 treatment in PD mice. Rows represent individual metabolites whose concentrations are represented as *Z* scores (*n* = 5–6 for each group), Columns represent groups. (A) Midbrain. (B) Striatum. (C) Cortex.

Based on the integrals of the signal, we determine the relative concentrations of distinct metabolites in the midbrain, striatum and cortex (Tables S2–S3). In the midbrain of PD mice, we found considerably greater levels of Lac, Ace, Cho, GABA, Myo, Cre, and reduced levels of Ala, Tau, Glu, and Gln in the midbrain of PD mice, while the disturbing levels of metabolites were changed to normal after FGF21 treatment (Figure 3; Table S1). After hierarchical clustering, numerous intriguing metabolite clusters emerged, including the Glu‐Gln‐GABA cycle, choline metabolism, and lactate metabolism (Figure 3A). In the striatum, higher amounts of Glu and decreased levels of GABA, Cho, and Lac were seen in the striatum in the PD group, but the metabolic pattern was restored in the FGF21 + MPTP group by boosting Cho, GABA, and reducing Glu after FGF21 treatment (Figure 3B; Table S2). In the cortex, FGF21 strongly recovers the alterations in Glu‐Gln‐GABA recycling, energy metabolism, and choline metabolism generated by MPTP injection (Figure 3C; Table S3).

### 3.3 FGF21 re‐structured gut microbiota profile in PD mice

**FIGURE 4:**
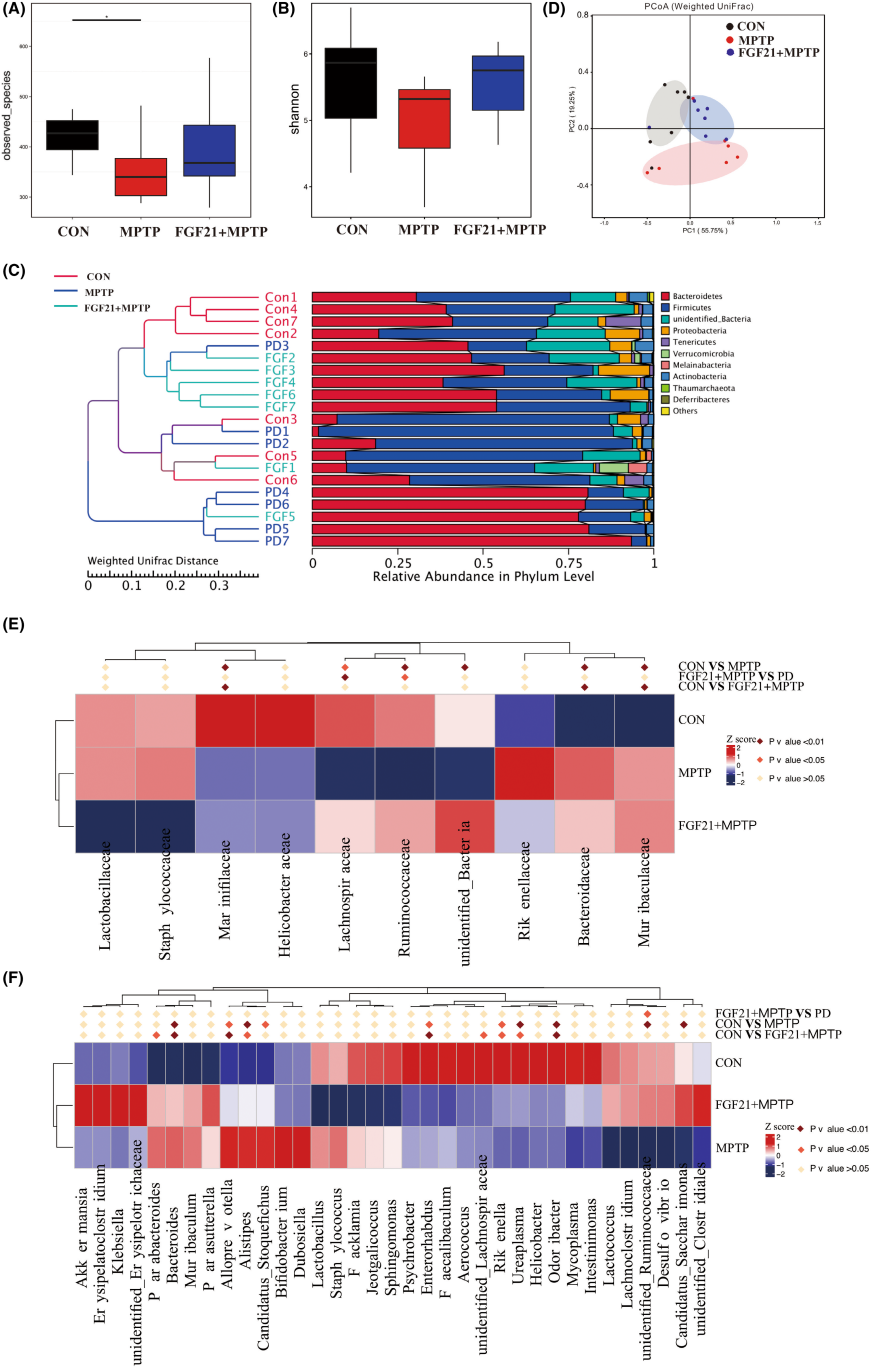
FGF21 modifies the dysbiosis of gut microbiotas induced by MPTP based on 16S rRNA sequencing. 16S rRNA sequencing‐predicted gut microbiota richness by the index observed species (A) and Shannon index (B). (C) Comparison of the fecal microbiota structures and distribution at the phylum level. (D) Principal coordinates analysis (PCoA) score plot. Relative abundances of gut microbiota at Family level (E) and Genus level (F). Columns represent individual microbiota whose concentrations are represented as *Z* scores. Red asterisks represent significant differences between groups after MetaStatic analysis (*n* = 7 for each group).

High‐throughput sequencing analysis was conducted to assess the impact of FGF21 on gut microbiota composition in PD mice. The Shannon Diversity Index and observed species showed that mice from the FGF21 + MPTP group were characterized by a higher richness than PD mice (Figure 4A,B). We further found that *Bacteroidetes* and *Firmicutes* were the most dominant phylum in all three groups. Significant phylum‐level changes in the microbiota communities were observed. MPTP injection significantly increased the abundance of *Bacteroidetes* and decreased the abundance of *Firmicutes* compared, while FGF21 treatment significantly increased the abundance of *Firmicutes* and decreased the abundance of *Bacteroidetes* compared with PD mice (Figure 4C). Moreover, the principal coordinate analysis (PCoA) plot revealed distinct clustering of microbiota composition between the CON, MPTP, and FGF21 + MPTP groups (Adonis *p* value = 0.002, Figure 4D), suggesting that FGF21 may inhibit the PD‐induced gut microbial dysbiosis.

Figure 4E profiles the response of microbiota diversity at the family level, increases in the abundances of *Bacteroidaceae* and *Muribaculaceae* and decreases abundances of *Ruminococcaceae* and *Lachnospiraceae* were identified in the PD mice compared with CON mice. Moreover, the abundances of certain SCFA‐producing bacteria (*Ruminococcaceae* and *Lachnospiraceae*) were significantly increased by FGF21 (Figure 4E). At the genus level, MPTP treatment significantly reduced the abundance of bacteria in the genus *Candidatus_Saccharimonas, unidentified_Ruminococcaceae, Odoribacter* and *Ureaplasma* (*p* < 0.01), while this reduction was blocked by FGF21 treatment (Figure 4F). These results suggested that FGF21 significantly modulated the colonic microbiota disturbance and enhanced the recovery of microbiota in PD mice.

**FIGURE 5:**
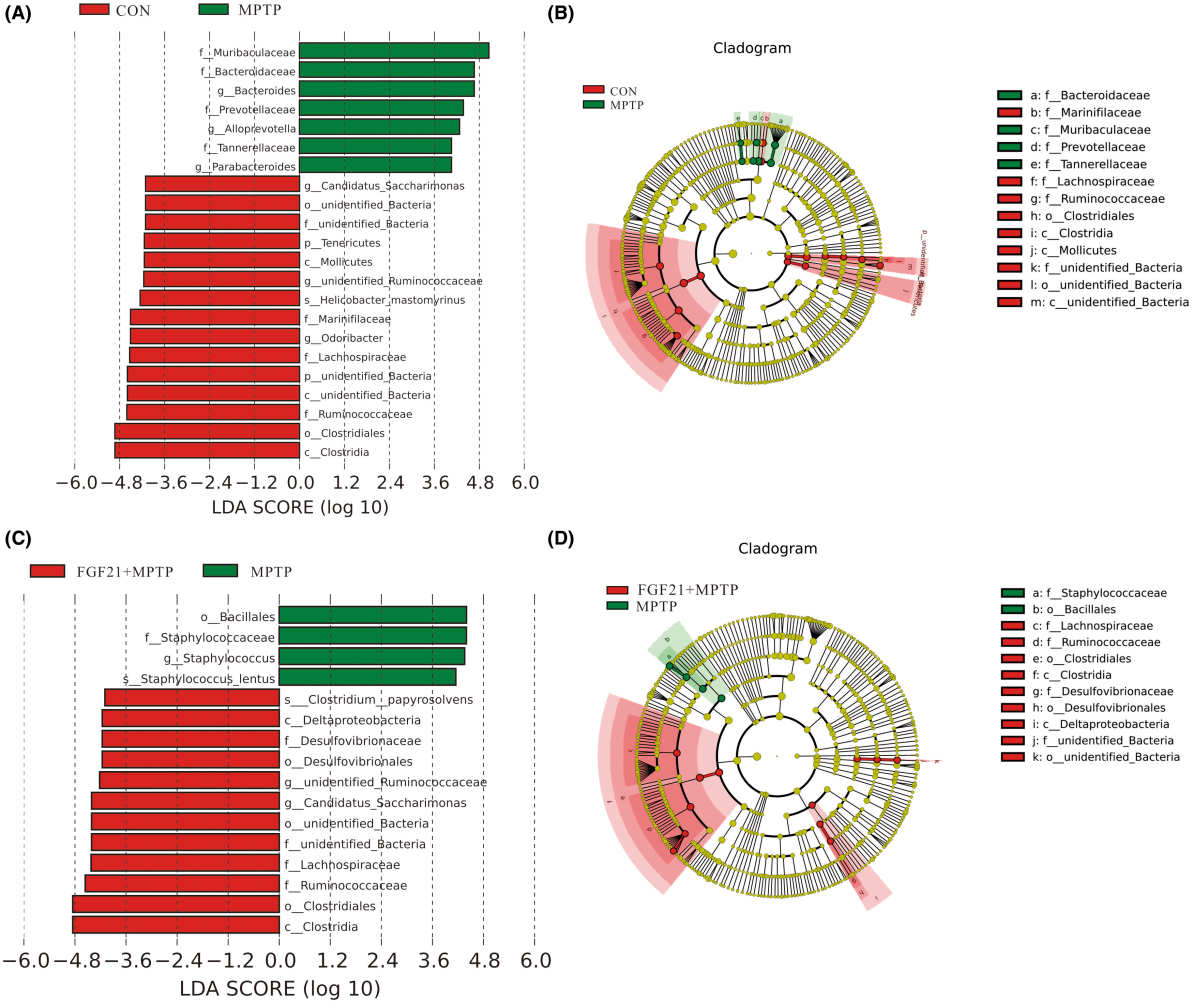
Linear discriminant analysis Effect Size (LEfSe) shows microbiome differences among 3 groups at various taxonomic levels. (A) LEfSe analysis with LDA score representing statistical bacterial differences in colonic microbiota between the PD (positive score) and CON groups (negative score). Functions meeting a linear discriminant analysis significant threshold >4 are shown. LDA: Linear discriminant analysis. (B**)** Cladogram using the LEfSe method indicating the phylogenetic distribution of colonic microbiota associated with PD and Con mice. Circles was used to indicate phylogenetic levels from phylum (innermost circle) to species (outermost circle) and circle diameter is proportional to the abundance of taxon. (C) LEfSe analysis with LDA score representing statistical bacterial differences in colonic microbiota between the PD (positive score) and FGF21 groups (negative score). (D) Cladogram using the LEfSe method indicating the phylogenetic distribution of colonic microbiota associated with PD and FGF21 participants.

LEfSe plots identified that 15 bacterial taxa displayed distinct relative abundances between CON and MPTP groups (LDA score >4.0, Figure 5A,B **)**. The abundance of the *Muribaculaceae* and *Bacteroidaceae* family was significantly higher in the PD group, while the abundance of *Clostridiales*, *Ruminococcaceae*, *Lachnospiraceae* was significantly higher in the CON group. Most significantly, the *Muribaculaceae* displayed the strongest correlation with PD disease, with the mean abundance being increased by 3 folds in MPTP groups compared to CON mice (Figure S2A). Compared with PD mice, the abundance of *Clostridiales*, *Ruminococcaceae*, and *Lachnospiraceae* was significantly enriched in the FGF21 group (LDA score >4, Figure 5D,E). More interestingly, the mean abundance of *Ruminococcaeae* family from the Clostridia class was increased by 2.6 folds in the FGF21 group compared with PD mice (Figure S2B).

### 3.4 FGF21 alleviates PD‐induced metabolic disorders in the colon

**FIGURE 6:**
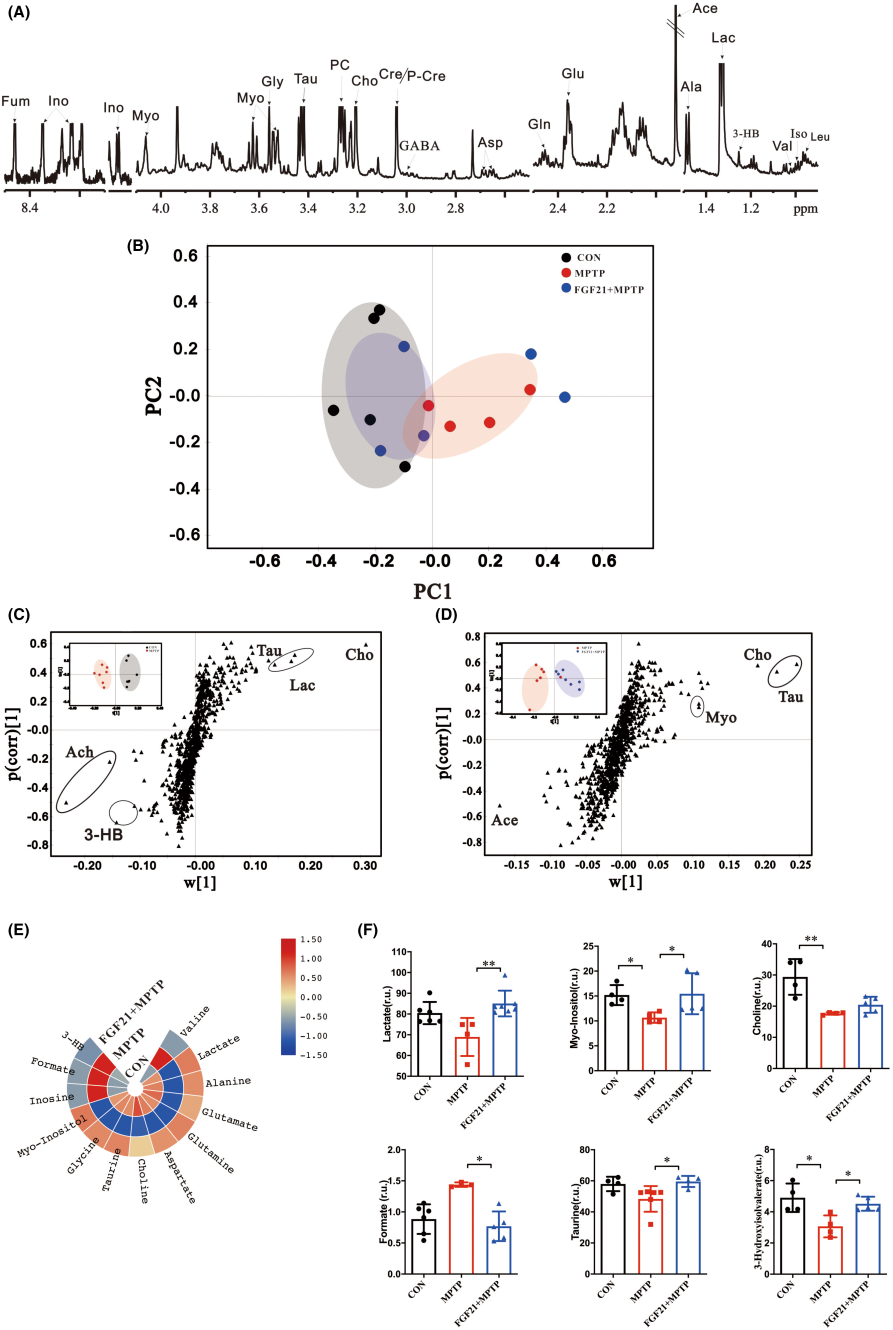
FGF21 abolishes the shifts of colonic metabolism in MPTP lesioned mice. (A) Representative 600 MHZ ^1^H NMR spectrum of the colon of CON, PD, and FGF21 mice. (B) PCA score plot of colon metabolites. (C) OPLS–DA score plot (inset) and OPLS–DA S‐plot of CON and PD mice. (D) OPLS–DA score plot (inset) and OPLS–DA S‐plot of PD mice after FGF21 treatment. (E) Colon metabolites whose concentrations are affected by PD disease and FGF21 treatment. (F) Metabolites significantly changed among the 3 groups. r.u. relative unit. Choline (*F*‐value = 12.09, df = 12, *p* = 0.0021, ANOVA test); Formate (*F*‐value = 9.67, df = 13, *p* = 0.0038, ANOVA test); Taurine (*F*‐value = 4.67, df = 13, *p* = 0.03, ANOVA test); 3‐HB (*F*‐value = 7.99, df = 12, *p* = 0.0085, ANOVA test); Data are mean ± s.e.m, *n* = 4–6 for each group. One‐way ANOVA analysis was conducted followed by a Tukey's multiple comparisons test. Data that do not exhibit a normal distribution was analyzed using non‐parametric Kruskal–Wallis test. Significant correlations are expressed as *p < 0.05; **p < 0.01; ***p < 0.001.

To test the hypothesis that FGF21 treatment influences brain functions through the gut microbiota–gut–brain metabolic pathway, we measured metabolites in the colon of mice after FGF21 injection. Typical ^1^H NMR spectra from the colon were displayed in Figure 6A. PCA score plot demonstrated that the metabolic profile of PD mice clustered distinctly from that of CON and FGF21‐treated mice (Figure 6B). This effect was more pronounced in OPLS–DA score plot (Figure 6C,D). The accompanying S plot also revealed that choline, taurine, lactate, and 3‐hydroxybutyrate (3‐HB) were responsible for the separation (Figure 6C,D). In addition, we observed significantly lower levels of lactate, choline, taurine, and 3‐HB in the colon of PD mice, compared with the CON group, while the reduced levels of metabolites were restored after treatment with FGF21 in PD mice (Figure 6E,F).

**FIGURE 7:**
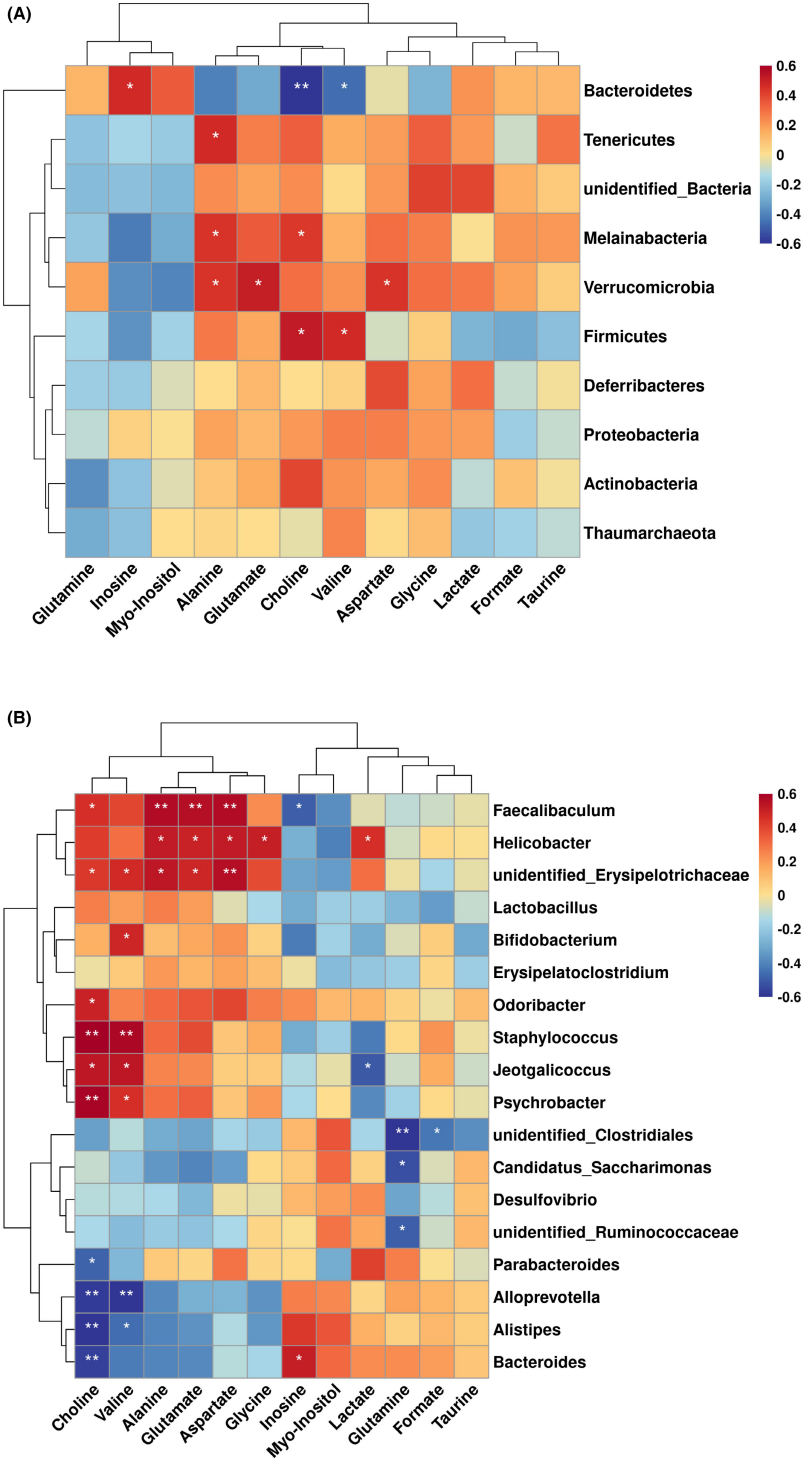
Spearman's correlation analysis on gut microbiota and colon metabolites. Correlation between gut microbiota and colon metabolites at the phylum level (A) and genus level (B). Color intensity suggests the association degree (Red: positive correlation; Blue: negative correlation). Significant correlations are expressed as *p < 0.05; **p < 0.01; ***p < 0.001.

To further explore the underlying effects of FGF21 on the microbiota–brain metabolic axis, relationships between colonic microbial abundance and colone metabolites were examined with Spearman correlation analysis (Figure 7). The findings showed a strong positive association between the relative abundance of *Firmicutes* choline and valine at the phylum level. In contrast, *Bacteroidetes* displayed a positive correlation with inosine in the colon but a negative correlation with choline and valine (Figure 7A). At the genus level, a positive correlation was discovered between *unidentified_Erysupelotrichacrae* and *Feacecalibaculum* with choline, glutamate, and aspartate. On the contrary, there was a negative association between the level of *Allpprevotella*, Alistipes, and *Bacteroides* and choline and valine (Figure 7B). In addition, a significant negative correlation was also identified between *unidentified_Ruminococcaceae*, *candidates_saccharimonas*, and glutamine (*p* < 0.05, Figure 7B), pointing to a potential role for this metabolic pathway in the neuroprotective effects of FGF21.

## 4 DISCUSSION

While many studies have indicated that gut–brain crosstalk was involved in the pathology of PD, few studies have focused on therapies in the gut microbiota–gut–brain metabolic axis. In the current study, we provided the first evidence that FGF21 treatment alleviated motor and cognitive function in PD mice via modulating gut microbiota and maintaining gut–brain metabolic homeostasis, benefiting from comprehensive investigations on neuronal function, gut microbiota profiling, brain‐region specific metabolic homeostasis, and metabolic profiling in the colon.

The neuroprotective effects of FGF21 have been shown to be of clinical value in treating neurodegenerative disease. Recently, we reported that peripheral administration of FGF21 ameliorates MPTP‐induced behavior deficits and neuroinflammation in mice.^19^ Other studies have indicated that FGF21 could alleviate neurological injury by modulating astrocyte‐neuron lactate shuttle, repairing brain mitochondrial damage, and ameliorating neuroinflammation.^15^, ^25^, ^26^ In the current study, mice receiving FGF21 treatment exhibited significantly higher spontaneous activity. Consistent with previous findings,^27^, ^28^ this study further proved that FGF21 treatment promotes functional recovery and alleviated neurodegeneration in PD mice. In addition, a notable finding of this study is that FGF21 is also able to ameliorate cognitive impairment in PD as demonstrated by the Y maze test. Thus, our results showed that FGF21 can not only improve motor function but also improve the non‐motor function, such as cognitive impairment. FGF21 could be an effective treatment option for patients with PD, leading to improvement in the recovery of both motor and non‐motor symptoms.

Previous studies have reported that administration of FGF21 and FGF21 analogs reduce bodyweight in animal models of obesity and in short‐term clinical trials.^29^ In contrast to earlier findings, however, we found that FGF21 increased body weight in PD mice model, this discrepancy may attribute to the different animal models. Unlike the previous studies, our study was conducted in a MPTP‐induced model, the body weight in model group was significantly decreased. Although MPTP‐induced mice model remains the most commonly used animal model of PD, this chemical lesioned animal model differs from the chronic progression characteristic of PD.^30^ If additional human studies provide evidence for a significant association between FGF21 expression and pathologic progression of PD, the results in study could help guide the design of clinical studies that evaluate the efficacy and safety of strategies such as FGF21 analogs treatment for improving motor and non‐motor deficits in patients with PD.

Epidemiological studies have found males with PD have significantly greater motor disorders and cognitive impairments compared with females.^31^ Though sex is a biological variable that may influence the effect of FGF21 on the brain, very little was found in the literature on the gender difference of FGF21 impacts on neurodegeneration disease. Previous studies reported that FGF21 affected micronutrient intake and energy metabolism both in male and female laboratory animals and the direct action on the neurons in the brain is required for this effect in both sexes,^32^, ^33^ suggesting that the effects of FGF21 on the brain are at least partially shared between male and female participants. Future research that examines the sex‐specific effects of FGF21, perhaps with a focus on brain energy metabolism may improve the early treatment of motor and cognitive dysfunction in PD.

Given the growing body of evidence linking metabolic homeostasis to neuronal function, and the fact that FGF21 is widely recognized as a metabolic regulator,^34^, ^35^ FGF21 could be involved in the neuronal function through modulating brain energy metabolism. Interestingly, a recent study reported that the effects of FGF21 on metabolic regulation can be mediated through mediating the cross‐talk between the liver and brain,^36^ while the potential role of FGF21 on metabolic homeostasis in the brain remains unknown. We examined the composition of metabolites in the brain of mice that had been treated with or without FGF21. Our results showed that the mice receiving FGF21 injection showed a region‐specific recovery from PD‐induced brain metabolic disorders, especially in the midbrain, striatum, and cortex. Here, we for the first time showed that FGF21 treatment for only 1 week completely reversed gut–brain metabolic imbalances in PD mice. We and others have demonstrated that brain region‐specific metabolic abnormity occurs in patients with PD and animal models.^9^, ^37^ In the present study, the over‐reactive glutamate–glutamine–GABA cycle and decreased taurine were observed in the midbrain of PD mice, while FGF21 treatment significantly restored the metabolic homeostasis by decreasing glutamate and increasing choline levels.

It has been reported that alterations of gut microbiota and neurotransmitter metabolism may be involved in the neuronal apoptosis in the striatum of PD mice.^38^ From the microbiota–gut–brain axis, changes in colonic microbiota may act as environmental triggers for neurodegeneration and behavior dysfunction in PD.^39^ More recently, the identified PD index based on gene sets from gut microbiota has shown to be a potential diagnostic biomarker of PD,^40^ which reinforces the notion that alteration in gut microbiota potentially participates in the pathology of PD. Here, we found that FGF21 treatment significantly increased the alpha diversity of colonic microbiota, suggesting an overall recovery in microbial status in PD mice after FGF21 treatment. Intriguingly, the mean abundance of the *Ruminococcaeae* family from the Clostridia class was increased by 2.6 folds in the FGF21 group compared with PD mice. Previous results showed that the *Ruminococcaceae* and *Lachnospiraceae* are among the main producers of butyrate, which could modulate brain metabolism and plasiticity.^41^, ^42^, ^43^ Our observations raise the possibility that the gut microbiota might contribute to the protective effects of FGF21 in PD.

As regards to the gut metabolic homeostasis, our results also showed that FGF21 promotes restoration of colon homeostasis in PD mice. Further multivariate analysis based on colon metabolic profile also identified significant difference in the level of lactate, choline, taurine and 3‐HB in FGF21 mice compared with CON mice. 3‐HB, also known as β‐hydroxybutyrate, is the most prevalent type of ketone in the human body. Recent publications indicate that 3‐hydroxybutyrate plays a neuroprotective role in PD mice model.^44^, ^45^ Taurine, one critical nutrient for neuron cells, has also been shown to improve neuron injuries in PD mice.^46^ In the present study, FGF21 significantly upregulated the level of 3‐HB and taurine, suggesting that these active metabolites may serve as signal molecules linking gut microbiota and brain metabolism and brain function.

To illustrate the connection between colonic microbial abundance and metabolic homeostasis, a correlation analysis was conducted between colonic microbial abundance and colon metabolites. At the genus level, we identified that the level of *Faecalibaculum* and *unidentified _Erysipelotrichaceae* was positively correlated with choline, valine, alanine, glutamate and aspartate, while the level of *Allpperavotella*, *Alistipes*, and *Bacteroides* was negatively correlated with choline and valine and glutamate (Figure 7B). Herein, the correlation analysis revealed that some altered gut microbiota was strongly correlated with altered choline metabolism, glutamate–glutamine cycle and branched‐chain amino acids in the colon. Previous studies have shown that some of the metabolites produced by the gut microbiota could help to maintain the gut metabolic homeostasis,^47^ which then contributes to maintaining the homeostasis and neuronal function of host through gut–brain aixs.^48^ We can infer that increased levels of choline, valine, and glutamate in the FGF21‐treated gut are possibly by modulating gut microbiota composition. And the alteration of gut metabolites could be one of the key regulators between gut microbiota community and brain metabolic homeostasis and behavior function in PD.

## 5 CONCLUSIONS

In summary, our findings support the hypothesis that FGF21 could modulate gut microbiota profile, restore region‐specific metabolic homeostasis and ameliorate behavior deficits in PD mice. Brain regional specific metabolic metabolism, such as choline metabolism, glutamate–glutamine cycle, and energy metabolism were involved in FGF21 protection. These findings also suggested a novel link between gut microbiota and neuronal function via the microbiota–gut–brain metabolic axis under the treatment of FGF21 in PD mice.

## AUTHOR CONTRIBUTIONS

Changwei Yang: Conceptualization, Writing‐Original draft preparation. Wuqiong Wang: Investigation, Data curation. Pengxi Deng and Xinyi Wang: Investigation. Lin Zhu: correlational analyses and visualization. Liangcai Zhao: Writing‐Reviewing and Editing. Chen Li: Methodology, Project administration. Hongchang Gao: Supervision, Funding acquisition. All the authors read and approved the final manuscript.

## CONFLICTS OF INTEREST STATEMENT

The authors declare no competing financial interests in this study.

## Supporting information


Appendix S1

## Data Availability

The data that support the findings of this study are available from the corresponding author upon reasonable request.
