# Supplementary material for: Fibroblast growth factor 21 ameliorates behavior deficits in Parkinson's disease mouse model via modulating gut microbiota and metabolic homeostasis
Source: CNS Neurosci Ther. 2023 Jun 19;29(12):3815–28. doi: 10.1111/cns.14302 (PMC10651963; doi:10.1111/cns.14302)
Supplement: Supplementary file 1 — Appendix S1 [file CNS-29-3815-s001.docx]

**Supplemental Material**

**Fibroblast growth factor 21 ameliorates behavior deficits in Parkinson’s disease mouse model via modulating gut microbiota and metabolic homeostasis**

Changwei Yang^1,2^, Wuqiong Wang^1^, Pengxi Deng^1^, Xinyi Wang^1^, Lin Zhu^2^, Liangcai Zhao^1^, Chen Li^1,3*^, Hongchang Gao^1,3^*

^1^ Institute of Metabonomics & Medical NMR, School of Pharmaceutical Science, Wenzhou Medical University, Wenzhou 325035, China;

^2^ School of Public health, Fujian Medical University, Fuzhou 350122, China;

^3^ Oujiang Laboratory (Zhejiang Lab for Regenerative Medicine, Vision and Brain Health), 18 Longwan District, 325000 Wenzhou, China

**^*^Correspondence:**

Hongchang Gao, Chen Li

E-mail: lichen2zh@163.com(C. Li); gaohc27@wmu.edu.cn (H.C. Gao);

**Method S1. *Colonic Microbiota DNA extraction and Sequencing***

Microbiota sequencing was conducted to determine the effects of FGF21 on PD-induced microbiota alteration. All DNA isolation, enrichment and sequencing were performed by Novogene (Beijing, China). Briefly, total genome DNA was extracted from colonic contents using CTAB/SDS method. DNA concentration and purity were monitored on 1% agarose gels. The V4 hypervariable regions (V4) of 16S rRNA genes were amplified using specific primers (515F-806R) and the indexing barcode was integrated. Sequencing libraries were generated using Ion Plus Fragment Library Kit (48 rxns, Thermo Scientific) following manufacturer's recommendations. At last, 400 bp/600 bp single-end reads were generated on an Ion S5TM XL platform. Single-end reads was assigned to samples based on their unique barcode and truncated by cutting off the barcode and primer sequence. Which are then further processed by removing reads of low quality and chimera sequences from the raw sequencing reads sets. On average 77.6 million high-quality reads per sample were generated for further analyses. OTU picking was performed by Uparse software (Uparse v7.0.1001). Sequences with ≥97% similarity were assigned to the same OTUs. For each representative sequence, the Silva Database (https://www.arb-silva.de/) was used based on Mothur algorithm to annotate taxonomic information. Multiple sequence alignment was conducted using the MUSCLE software (Version 3.8.31, <http://www.drive5.com/muscle/)>. OTUs abundance information was normalized using a standard of sequence number corresponding to the sample with the least sequences.

Alpha diversity and Beta diversity analysis were used to evaluate the effects of FGF21 on colonic microbiota. Alpha diversity was assessed through 6 indices: Observed-species, Chao1, Shannon, Simpson, ACE, Good-coverage, was calculated with QIIME (Version 1.7.0) and displayed with R software (Version 2.15.3). Beta diversity on both weighted and unweighted unifrac distance matrices were calculated by QIIME software [[1](#_ENREF_1)] (Version 1.7.0). Principal Coordinate Analysis (PCoA) was performed to get principal coordinates and to visually explore sample separation and similarity. PCoA analysis was displayed by WGCNA package, stat packages and ggplot2 package in R software (Version 2.15.3). Unweighted Pair-group Method with Arithmetic Means (UPGMA) Clustering was performed as a type of hierarchical clustering method to interpret the distance matrix using average linkage and was conducted by QIIME software (Version 1.7.0).

In addition, we also applied linear discriminant analysis effect size (LEfSe) analysis [[2](#_ENREF_2)] to identify taxa or pathways differentially abundant between CON, PD and FGF21 groups. This method first uses the non-parametric factorial Kruskal-Wallis sum-rank test to detect features with significant differential abundance and then uses linear discriminant analysis (LDA) to calculate the effect size of each feature.

**Table S1: Metabolite alterations in the midbrain of CON, MPTP and FGF21+MPTP group**

| **Metabolite** | **CON^a^** | **MPTP^b^** | **FGF21+MPTP^c^** |
| --- | --- | --- | --- |
| **Lactate** | 76.54±3.80 | 80.04±2.09 | 76.31±4.11↓^#^ |
| **Acetate** | 10.98±1.19 | 12.63±1.41↑* | 11.03±0.62↓^#^ |
| **Choline** | 45.23±2.81 | 51.84±0.49↑** | 48.06±1.32↓^#^ |
| **Alanine** | 9.36±0.44 | 8.08±0.69↓** | 8.86±0.53 |
| **Taurine** | 40.05±2.88 | 26.39±4.36↓** | 29.29±0.53 |
| **Glutamate** | 27.40±0.78 | 24.79±0.27↓*** | 27.41±0.08↑^###^ |
| **Glutamine** | 32.11±0.31 | 28.56±0.25↓** | 30.80±1.26↑^#^ |
| **GABA^d^** | 20.98±0.86 | 25.11±0.81↑** | 21.88±2.42↓^#^ |
| **Myo^e^** | 30.28±3.08 | 38.92±1.29↑*** | 34.94±1.61↓^#^ |
| **Creatine** | 43.63±1.81 | 51.31±1.15↑*** | 47.47±0.98↓^##^ |
| **3-HB^f^** | 15.16±1.13 | 18.40±2.38 | 16.18±0.91 |
| **NAA^g^** | 32.24±1.64 | 31.48±0.85 | 33.97±1.50 |
| **Aspartate** | 7.08±1.51 | 9.08±0.80 | 7.91±0.68 |
| **Glycine** | 9.37±0.96 | 11.28±0.43 | 10.59±0.82 |
| **Anserine** | 11.86±0.96 | 11.06±0.53 | 11.25±0.62 |
| **P-Cre^h^** | 33.08±2.11 | 36.32±1.45 | 35.24±1.31 |
| **IMP^i^** | 1.82±0.39 | 2.00±0.52 | 2.51±0.25 |
| **AMP^j^** | 2.24±0.29 | 2.26±0.15 | 1.91±0.09 |
| **Inosine** | 2. 97±0.20 | 2.80±0.13 | 2.54±0.14 |
| **Leucine** | 11.80±0.96 | 10.67±1.25 | 11.48±1.80 |
| **Isoleucine** | 2.26±0.24 | 2.17±0.23 | 2.15±0.11 |

* means the difference between CON mice and MPTP mice; # means the difference between MPTP and FGF21+MPTP group. * P < 0.05, ** P < 0.01, *** P < 0.001; # P < 0.05, ## P < 0.01, ### P < 0.001. Data were expressed as the mean ± SEM (n=6-10 for each group). ^a^ control mice; ^b^ MPTP-induced mice; ^c^ FGF21-treated mice; ^d^ γ-Aminobutyric acid; ^e^ myo-inositol; ^f^ 3-hydroxybutyrate; ^g^ N-acetylaspartate; ^h^ Creatine phosphate；^I^ inosine monophosphate; ^j^Adenosine monophosphate

**Table S2: Metabolite alterations in the stratum of CON, MPTP and FGF21+MPTP mice**

| Metabolite | **CON^a^** | **MPTP^b^** | **FGF21+MPTP^c^** |
| --- | --- | --- | --- |
| Lactate | 76.73±2.96 | 62.49±3.75↓** | 71.50±3.87↑^#^ |
| Glutamate | 22.01±0.30 | 25.89±2.00↑* | 22.56±0.27↓^#^ |
| Choline | 15.66±0.70 | 14.05±1.52↓** | 14.96±1.07↑^#^ |
| GABA^d^ | 19.16±1.00 | 14.90±0.78↓*** | 16.14±0.51↑^#^ |
| Myo^e^ | 24.44±1.15 | 21.54±3.83 | 25.61±0.30↑^#^ |
| P-Cre^f^ | 29.13±2.06 | 31.98±2.08 | 30.34±0.62 |
| Glycine | 9.02±0.67 | 7.90±0.60 | 8.93±1.49 |
| Anserine | 12.54±3.16 | 20.90±1.65↑** | 14.57±3.16↓^##^ |
| Glutamine | 30.60±2.47 | 34.29±6.21 | 31.53±1.75 |
| Aspartate | 7.07±0.52 | 8.17±2.79 | 5.99±0.48 |
| NAA^g^ | 29.46±2.88 | 22.55±7.86 | 27.54±2.08 |
| 3-HB^h^ | 9.67±2.47 | 7.12±0.70 | 9.47±1.92 |
| Alanine | 11.49±2.12 | 9.49±0.18 | 10.06±0.57 |
| Acetate | 9.95±0.62 | 10.42±2.45 | 9.34±0.66 |
| IMP^i^ | 1.11±0.22 | 0.84±0.20 | 1.17±0.18 |
| AMP^j^ | 2.59±0.29 | 2.16±0.23 | 2.03±0.34 |
| Inosine | 3.34±0.15 | 3.92±0.22 | 3.77±0.13 |
| Leucine | 11.13±0.56 | 11.86±0.89 | 11.51±0.70 |
| Isoleucine | 2.09±0.07 | 2.64±0.51 | 2.18±0.18 |

* means the difference between CON mice and MPTP mice; # means the difference between MPTP and FGF21+MPTP group.* P < 0.05, ** P < 0.01, *** P < 0.001; # P < 0.05, ## P < 0.01. Data were expressed as the mean ± SEM (n=6-10 for each group). ^a^ control mice; ^b^ MPTP-induced mice; ^c^ FGF21-treated mice; ^d^ γ-Aminobutyric acid; ^e^ myo-inositol; ^f^ 3-hydroxybutyrate; ^g^ N-acetylaspartate; ^h^ Creatine phosphate；^I^ inosine monophosphate; ^j^Adenosine monophosphate

**Table S3: Metabolite alterations in the cortex of CON, MPTP and FGF21+MPTP mice**

| **Metabolite** | **CON^a^** | **MPTP^b^** | **FGF21+MPTP^c^** |
| --- | --- | --- | --- |
| **Lactate** | 85.25±1.71 | 76.24±1.20↓*** | 81.49±1.75↑^##^ |
| **Alanine** | 9.71±0.06 | 10.78±0.89↑*** | 9.86±0.41↓^###^ |
| **NAA^d^** | 36.03±0.63 | 39.27±2.07↑* | 35.91±1.58↓^##^ |
| **Acetate** | 7.92±0.3 | 8.99±0.62↑* | 7.85±0.66↓^#^ |
| **Aspartate** | 17.94±0.57 | 20.59±1.91↑** | 17.37±0.54↓^###^ |
| **Glutamate** | 27.96±0.89 | 33.12±2.23↑*** | 30.01±0.69↓^###^ |
| **Glutamine** | 35.09±1.36 | 41.42±10.69↑** | 36.16±1.42 |
| **GABA^e^** | 18.21±1.52 | 15.24±0.40↓*** | 17.17±1.18↑^#^ |
| **Choline** | 13.85±1.29 | 11.00±0.96↓** | 12.92±1.06↑^#^ |
| **3-HB^f^** | 15.70±3.36 | 10.69±2.85 | 13.80±1.32 |
| **Taurine** | 33.91±1.35 | 37.68±1.99↑* | 33.76±1.39↓^#^ |
| **Myo^g^** | 24.37±1.59 | 26.06±1.58 | 24.21±1.23 |
| **Creatine** | 43.73±2.82 | 44.65±2.90 | 43.25±1.72 |
| **Glycine** | 8.23±0.57 | 7.40±0.68 | 8.50±1.19 |
| **Anserine** | 12.85±0.64 | 14.57±3.39 | 13.54±0.79 |
| **P-Cre^h^** | 30.85±1.75 | 30.25±2.77 | 31.72±1.67 |
| **IMP^i^** | 2.59±0.74 | 2.82±0.63 | 2.80±0.32 |
| **AMP^j^** | 1.81±0.22 | 1.01±0.29 | 1.53±0.15 |
| **Inosine** | 2.43±0.30 | 1.41±0.34 | 2.33±0.36 |
| **Leucine** | 10.38±0.46 | 10.93±0.64 | 10.71±0.71 |
| **Isoleucine** | 2.14±0.31 | 1.90±0.11 | 2.22±0.27 |

* means the difference between CON mice and MPTP mice; # means the difference between MPTP and FGF21+MPTP group. * P < 0.05, ** P < 0.01; # P < 0.05, ## P < 0.01. Data were expressed as the mean ± SEM (n=6-10 for each group). ^a^ Control mice; ^b^ MPTP-induced PD mice; ^c^ FGF21-treated mice; ^d^ N-acetylaspartate; ^e^ γ-Aminobutyric acid; ^f^ 3-hydroxybutyrate; ^g^ myo-inositol; ^h^ Creatine phosphate; ^i^ inosine monophosphate; ^j^ Adenosine monophosphate

**Figure list**

**

**

**Fig S1 O-PLS-DA score plots and corresponding VIP plots obtained from NMR-based metabolomics of CON, PD and FGF21 mice in different brain regions: (A-B)** Midbrain. (**C-D)** Striatum. (**E-F)** Cortex. Metabolite assignment: lactate (Lac), 1.33 ppm; alanine (Ala), 1.48 ppm; acetate (Ace), 1.92 ppm; glutamine (Gln), 2.15 and 3.76 ppm; glutamate (Glu), 2.46 and 3.76 ppm; 4-Aminobutyrate (GABA), 2.30 ppm; creatine (Cre), 3.04 ppm; taurine (Tau), 3.26 and 3.43 ppm; Myo-inositol (Myo), 3.53, 3.63 and 4.06 ppm; AMP, 6.10 and 8.58 ppm; inosine (Ino), 8.35 ppm.

**
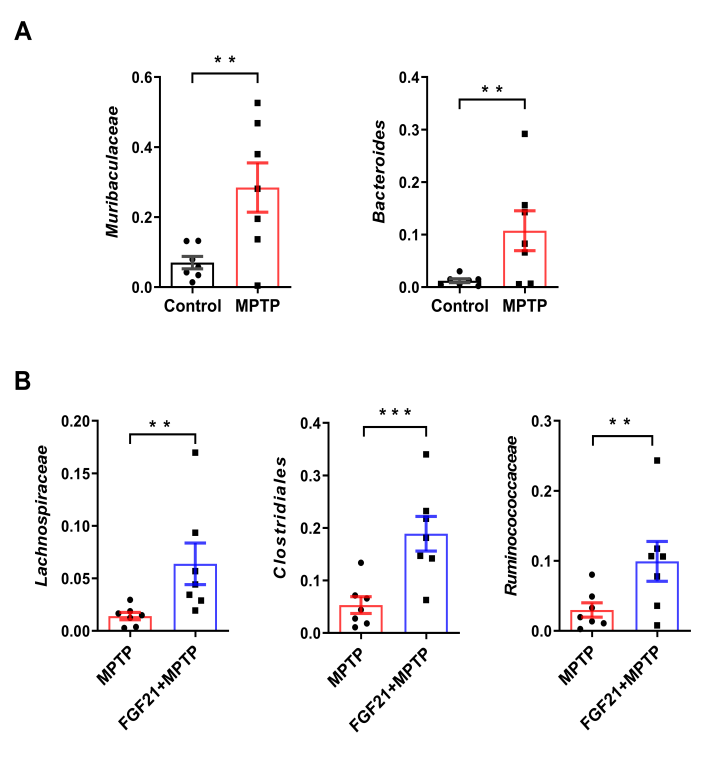
**

**Fig S2 Relative abundance of selected microbiota among three different groups.** (**A**) The relative abundance of *Muribaculaceae* and *Bacteroides* was significantly higher in the PD group compared with CON mice. **P < 0.01. (B) The relative abundance of Clostriadiales, Lachnospiraceae and Ruminococcaceae in FGF21 and PD mice. n=7 for each group. **P < 0.01, ***P < 0.001.

**References:**

1. Caporaso JG, Kuczynski J, Stombaugh J, Bittinger K, Bushman FD, Costello EK, Fierer N, Peña AG, Goodrich JK, Gordon JI, et al: **QIIME allows analysis of high-throughput community sequencing data.** *Nat Methods* 2010, **7:**335-336.

2. Lin CH, Chen CC, Chiang HL, Liou JM, Chang CM, Lu TP, Chuang EY, Tai YC, Cheng C, Lin HY, Wu MS: **Altered gut microbiota and inflammatory cytokine responses in patients with Parkinson's disease.** *J Neuroinflammation* 2019, **16:**129.
